# Improved RT-DETR and its application to fruit ripeness detection

**DOI:** 10.3389/fpls.2025.1423682

**Published:** 2025-02-27

**Authors:** Mengyang Wu, Ya Qiu, Wenying Wang, Xun Su, Yuhao Cao, Yun Bai

**Affiliations:** ^1^ School of Physics and Electronic Engineering, Jiangsu Normal University, Xuzhou, China; ^2^ School of Electrical Engineering and Automation, Jiangsu Normal University, Xuzhou, China; ^3^ School of Computer Science and Technology, Wuhan University of Science and Technology, Wuhan, Hubei, China; ^4^ College of International Studies, National University of Defense Technology, Nanjing, China

**Keywords:** RT-DETR, PConv, Rep Block, EMA, crop maturity detection

## Abstract

**Introduction:**

Crop maturity status recognition is a key component of automated harvesting. Traditional manual detection methods are inefficient and costly, presenting a significant challenge for the agricultural industry.

**Methods:**

To improve crop maturity detection, we propose enhancements to the Real-Time DEtection TRansformer (RT-DETR) method. The original model's Backbone structure is refined by: HG Block Enhancement: Replacing conventional convolution with the Rep Block during feature extraction, incorporating multiple branches to improve model accuracy. Partial Convolution (PConv): Replacing traditional convolution in the Rep Block with PConv, which applies convolution to only a portion of the input channels, reducing computational redundancy. Efficient Multi-Scale Attention (EMA): Introducing EMA to ensure a uniform distribution of spatial semantic features within feature groups, improving model performance and efficiency.

**Results:**

The refined model significantly enhances detection accuracy. Compared to the original model, the average accuracy (mAP@0.5) improves by 2.9%, while model size is reduced by 5.5% and computational complexity decreases by 9.6%. Further experiments comparing the RT-DETR model, YOLOv8, and our improved model on plant pest detection datasets show that our model outperforms others in general scenarios.

**Discussion:**

The experimental results validate the efficacy of the enhanced RT-DETR model in crop maturity detection. The improvements not only enhance detection accuracy but also reduce model size and computational complexity, making it a promising solution for automated crop maturity detection.

## 1 Introduction

As a nation renowned for its vast agricultural output, crops assume a paramount significance in human production and livelihood. Historically, harvesting has predominantly relied on manual labor, a practice notorious for its resource intensive nature and sluggish efficiency. Consequently, automatic harvesting has emerged as a progressive alternative. Presently, both domestic and international research endeavors pertaining to automatic crop harvesting predominantly center around image recognition, positioning, and picking (Bai et al., 2023). However, the accurate detection of crop maturity levels stands out as a pivotal facet for enhancing both harvesting efficiency and subsequent storage practices.

Most traditional computer vision methods employed to address the crop maturity level challenge are based on machine learning approaches such as K Neareat Neighbors (KNN) (Guo et al., 2003), Supported Vector Machine (SVM) (Wang and Hu, 2005), and Artificial Neural Network (ANN) (Agatonovic-Kustrin and Beresford, 2000). Semary et al. (2014) utilized support vector machines and Principal Component Analysis (PCA) to identify texture and color features from both HSV and RGB color spaces, achieving an accuracy of 92% in detecting surface defects in samples. Moallem et al. (2017) employed SVM, ANN and KNN as machine learning techniques to respectively discern plant decay levels, with the SVM classifier yielding the highest accuracy score of 92.5%. Although the aforementioned traditional machine learning methods have proven effective in crop maturity detection, they are inherently reliant on the characteristics of the studied objects and are subject to certain limitations.

Deep learning-based methodologies have gained significant traction in agricultural applications. These models possess the ability to automatically extract pertinent features devoid of human intervention. Suharjito et al. (2021) employed a MobileNetV1-based framework to classify oil palm into six distinct grades according to predefined criteria, achieving an accuracy rate of 81.1%. Gai et al. (2023) introduced the DenseNet architecture to pinpoint the locations of cherry fruits across varying ripeness levels utilizing the YOLOv4 network. Chen et al. (2022) leveraged YOLOv5 for initial target identification, followed by the integration of the saliency map into the ResNet34 network to ascertain fruit ripeness, ultimately attaining a detection accuracy of 95.07%. Single Shot MultiBox Detector (SSD) (Tan et al., 2020) is renowned for its real-time multi-scale object detection capabilities, and Ananthanarayana et al. (2020) proposed a methodology employing the SSD network in conjunction with MobileNetV2 (Sandler et al., 2018) for fruit ripeness detection, achieving a detection accuracy of 62%. A pivotal stage in target detection algorithm involves screening anchor frames based on the Non-Maximum Suppression(NMS) algorithm, however, the hyper-parameters of NMS, such as the Intersection over Union (IOU) threshold, and the scoring thresholds have a great impact on the accuracy and speed of the detector, which can lead to performance bottlenecks and need to be manually adjusted. While the advent of Real-Time DEtection TRansformer (RT-DETR) (Lv et al., 2023) has bolstered accuracy, its end-to-end structure markedly amplifies model memory and inference time.

Drawing upon the aforementioned challenges, we curated a collection of plant images sourced from the Internet. Employing image augmentation techniques such as flipping and panning, we augmented the dataset to forestall overfitting. With a focus on expediting neural network inference, we built upon the RT-DETR model by integrating Rep Block, inspired by RepVGG, into the Backbone component of the original model to enhance average accuracy. Furthermore, we incorporated the Partial Convolution (PConv) concept introduced by FasterNet (Chen et al., 2023), which strikes a favorable balance between speed and accuracy. Specifically, we replaced the regular convolution within the Rep Block with PConv to streamline computational efforts. Additionally, we introduced Efficient Multi-Scale Attention (EMA) mechanism subsequent to the Stem module of the Backbone. A novel cross-space learning method was employed to reshape a portion of the channel dimensions into batch dimensions, facilitating the grouping of channel dimensions into multiple sub-features. This strategy ensures uniform distribution of spatial semantic features across each feature group, yielding a discernible improvement in model parameters. The refined RT-DETR model significantly diminishes both model size and computational complexity while concurrently enhancing the accuracy of plant image maturity detection.

## 2 Overview of RT-DETR

In the domain of fruit detection, the YOLO series models have been extensively utilized due to their high detection efficiency and strong generalization capabilities. Numerous subsequent studies have proposed various improvement strategies to further enhance the performance of these models. For instance, Edy and Suharjito (2023) integrated the YOLOv4-Tiny model with a genetic algorithm to optimize the detection of oil palm ripeness, resulting in an improvement in the model’s mean Average Precision (mAP) by 0.1% and 0.5%, respectively. Similarly, Xu et al. (2024) introduced the RFAConv module to enhance the feature extraction capabilities of the core network, achieving a detection accuracy of 93.16%. While YOLO models offer significant advantages in terms of inference speed and lightweight design, the RT-DETR model demonstrates superior robustness and detection performance in complex scenarios. Specifically, the global feature modeling capability of RT-DETR allows it to effectively capture relationships between objects, providing a notable accuracy advantage in detecting fruit clusters where multiple targets are closely packed. This highlights the potential of RT-DETR to address challenges associated with complex detection tasks more effectively than traditional YOLO-based approaches.

**Figure 1:**
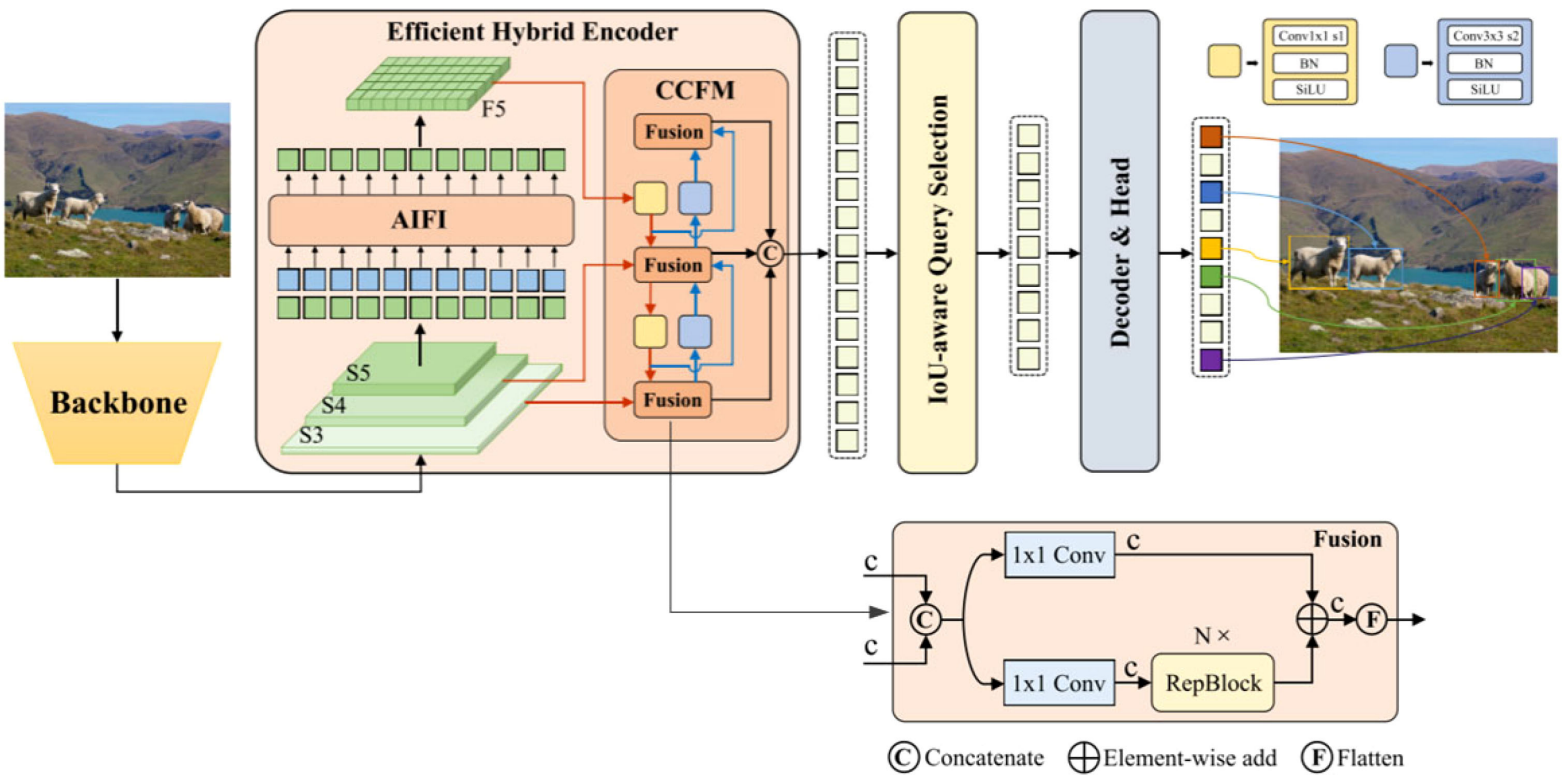
RT-DETR architecture (Lv et al., 2023).

RT-DETR represents a robust real-time end-to-end processor, seamlessly integrating intra- and cross-scale fusion through the utilization of Vision Transformers to effectively process multiscale features. RT-DETR leverages CNN architecture for its backbone network and employs a hybrid encoder for its encoder module. Notably, the decoder segment of RT-DETR incorporates a multi-layer Transformer decoder, affording the flexibility to adjust inference speed by employing different decoder layers without necessitating re-training. An overview of the model architecture is depicted in **Figure 1**.

As illustrated in **Figure 1**, the features extracted from the last three levels {S3, S4, S5} of the backbone network serve as inputs to the encoder. The hybrid encoder facilitates the transformation of multiscale features into a sequential representation of image features, achieved through Attention-based Intra-scale Feature Interaction (AIFI) (Chen et al., 2022) and CNN-based Cross-scale Feature Fusion (CCFF). An IoU-aware query mechanism is employed to pick a constant range of photograph features, which are in consequence utilized as the preliminary object query for the decoder. Subsequently, the decoder iteratively refines the object query, culminating in the era of prediction frames and related self assurance scores.

## 3 Improved RT-DETR model

**Figure 2:**
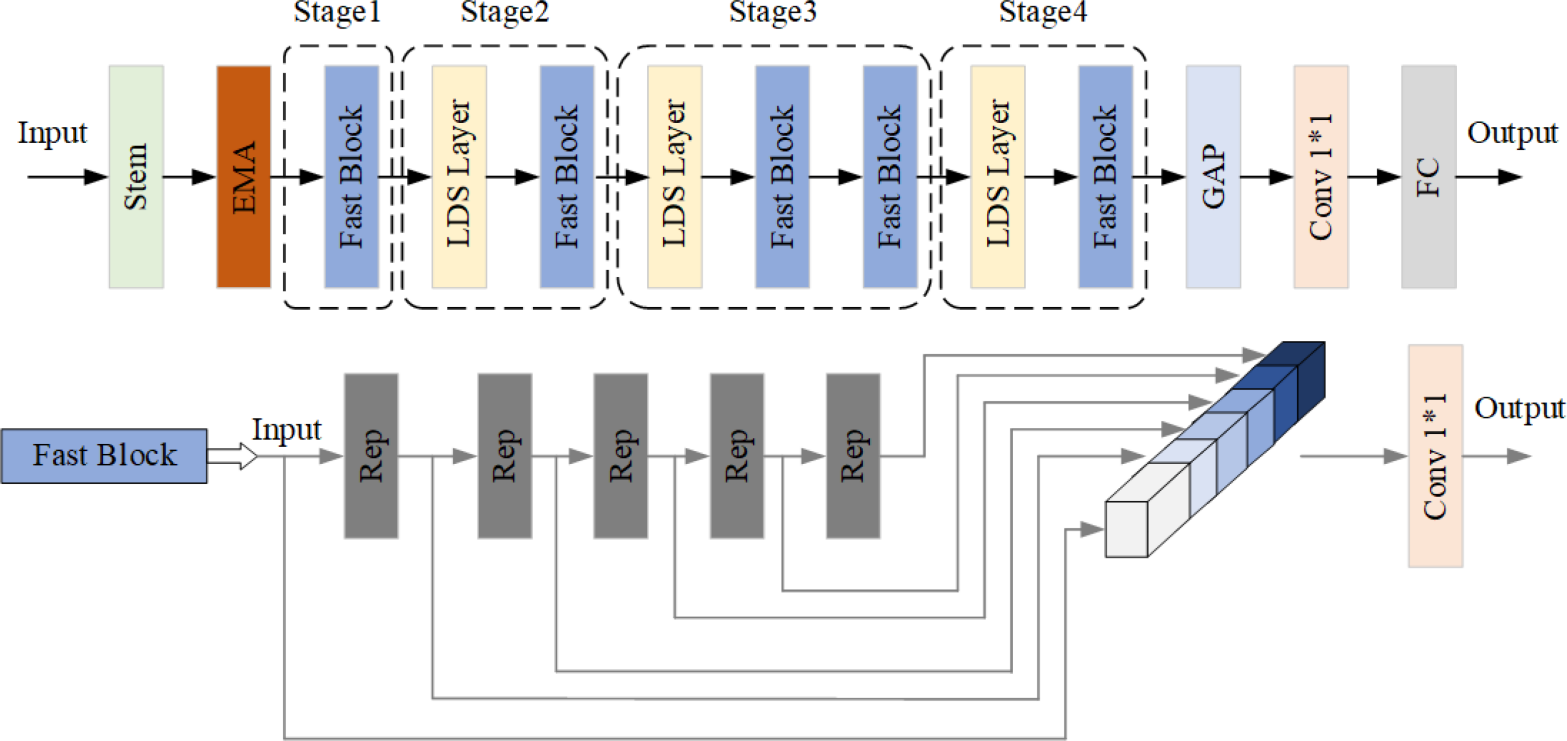
Improved backbone structure.

Building upon the RT-DETR-R18 model, this study enhances the backbone network, PP-HGNet, by introducing several modifications. Firstly, the Efficient Multi-Scale Attention (EMA) mechanism is integrated after the Stem block to reconfigure select channel dimensions into batch dimensions, thereby circumventing dimensionality reduction through conventional convolution and mitigating subsequent computational burdens. Furthermore, enhancements are made to the HG Block of the original backbone network by substituting the conventional 3×3 convolution with the Rep Block. This adjustment enables the incorporation of multiple parallel branches, augmenting the model’s characterization capabilities and bolstering accuracy. Additionally, the Rep Block’s ordinary convolution is replaced with Partial Convolution, which effectively manages computational overhead without significantly compromising accuracy. The refined Backbone network is visually depicted in **Figure 2**.

### 3.1 Rep Block

**Figure 3:**
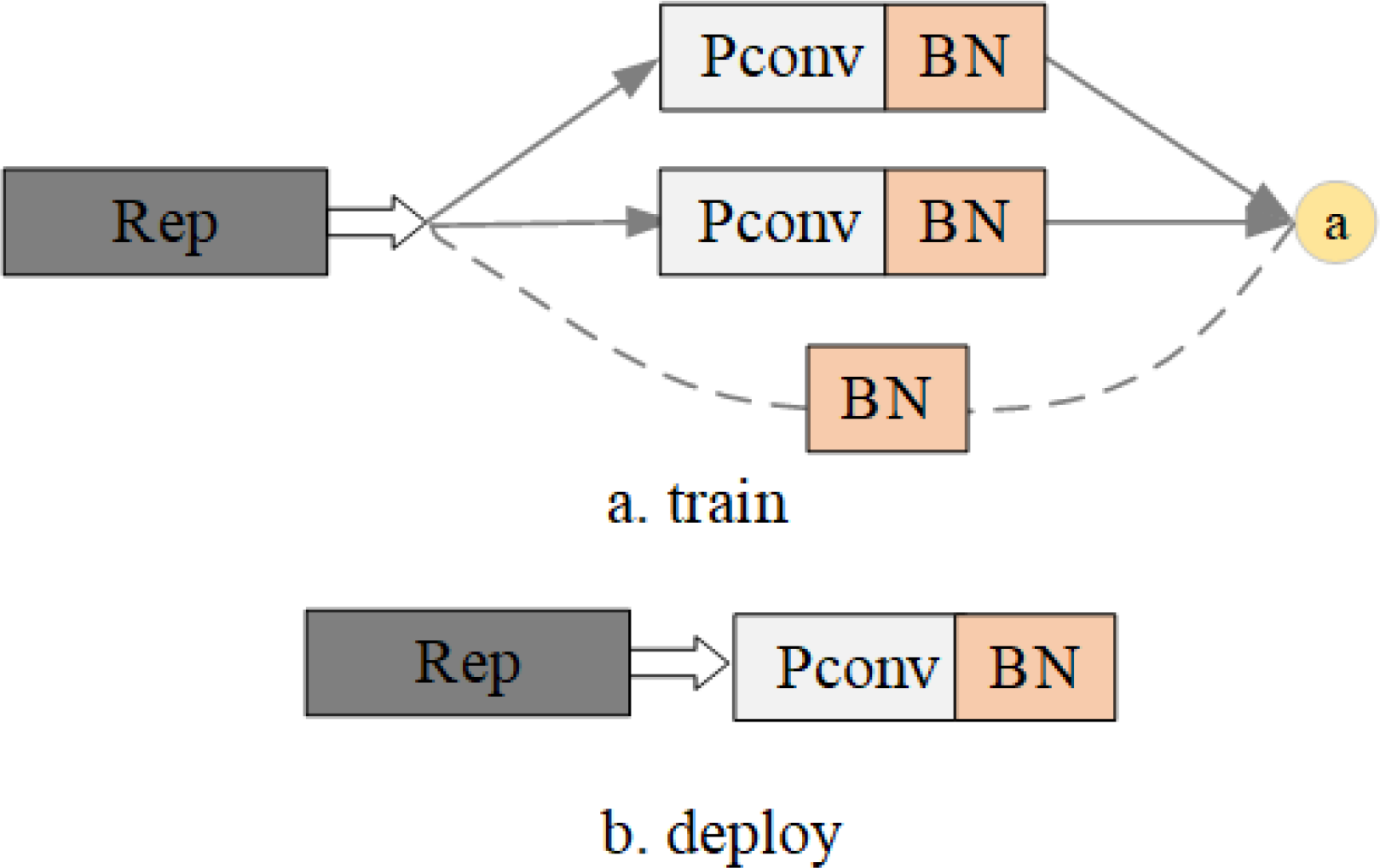
Rep Block structure.

RepVGG exhibits an inference time profile akin to VGG (Sengupta et al., 2019), featuring a composition comprising solely of a sequence of 3×3 Rep Blocks and Rectified Linear (ReLU) units, thereby surpassing other convolutional neural networks in performance. During training, the model adopts a multi-branching structure for the Rep Blocks, which subsequently transitions into a unidirectional model structure for inference. As delineated in **Figure 3**, Figure a depicts the network structure utilized during RepVGG training, whereas Figure b illustrates the network structure employed for inference. While the multi-branch structure yields higher accuracy, during inference, hardware resources are obligated to compute the outcomes of each branch individually, with the rapid branch necessitating a wait period for the completion of other branches’ calculations before proceeding with further fusion. Consequently, the hardware’s computational power remains underutilized. Hence, the conversion of the multi-branch structure into a single-branch configuration prior to inference becomes imperative.

The initial phase involves the construction of the training block. ResNet introduces a shortcut branch to capture the information flow as y=x+f(x) , employing a residual block to learn f . When the dimensions of x and f(x) are mismatched, the modeling formula adapts to y=g(x)+f(x) , where g(x) adjusts the number of channels via a 1×1 convolution. Drawing inspiration from ResNet, multiple branches are stacked to compose the training block: the information flow during training is modeled as y=x+f(x)+g(x) . The pinnacle department elements a 3×3 convolution for function extraction, the center department employs a 1×1 convolution for characteristic smoothing, and the remaining department conducts a panning operation.

Subsequently, We need to transform the training block into a 3×3 convolutional layer for the inference:

In the initial step, the convolutional operators and Batch Normalization (BN) operators across all three branches are merged to serve as convolutional operators. $W^{\left(3\right)}\in R^{C_{2}*C_{1}*3*3}$ denotes the convolutional kernel of the 3×3 branch, $W^{\left(1\right)}\in R^{C_{2}*C_{1}}$ signifies the kernel of the 1×1 branch, $C_{1}$ represents the input channel, and $C_{2}$ signifies the output channel. $\mu^{\left(3\right)}$ , $\sigma^{\left(3\right)}$ , $\gamma^{\left(3\right)}$ , and $\beta^{\left(3\right)}$ are employed to denote the cumulative mean, standard deviation, learning scaling factor, and bias of the BN layer. Furthermore, $\mu^{\left(1\right)}$ , $\sigma^{\left(1\right)}$ , $\gamma^{\left(1\right)}$ , $\beta^{\left(1\right)}$ denotes the parameters of the BN layer after the 1×1 convolution, and $\mu^{\left(0\right)}$ , $\sigma^{\left(0\right)}$ , $\gamma^{\left(0\right)}$ , $\beta^{\left(0\right)}$ denotes the parameters of the constant branch. Let $M^{\left(1\right)}\in R^{N*C_{1}*H_{1}*W_{1}}$ and $M^{\left(2\right)}\in R^{N*C_{2}*H_{2}*W_{2}}$ represent the input and output. If $C_{1}=C_{2},W_{1}=W_{2},H_{1}=H_{2}$ , then:


(1) $$\begin{array}{l} {\text{M}}^{\left(2\right)}=bn({\text{M}}^{\left(1\right)}*{\text{W}}^{\left(3\right)},\mu^{\left(3\right)},\sigma^{\left(3\right)},\gamma^{\left(3\right)},\beta^{\left(3\right)})\\ \;+bn({\text{M}}^{\left(1\right)}*{\text{W}}^{\left(1\right)},\mu^{\left(1\right)},\sigma^{\left(1\right)},\gamma^{\left(1\right)},\beta^{\left(1\right)})\\ \;+\;bn({\text{M}}^{\left(1\right)}*{\text{W}}^{\left(0\right)},\mu^{\left(0\right)},\sigma^{\left(0\right)},\gamma^{\left(0\right)},\beta^{\left(0\right)}) \end{array}$$

bn represents the BN function during inference, $\forall 1\leq i\leq C_{2}$


(2) $$bn{\left(\text{M},\mu ,\sigma ,\gamma ,\beta \right)}_{:,i,:,:}=({\text{M}}_{:,i,:,:}-\mu_{i})\frac{\gamma_{i}}{\sigma_{i}}+\beta_{i}$$

Transforming each BN layer along with its preceding convolutional layers into a convolution with bias vectors, $\left\{W^{\prime },b^{\prime }\right\}$ represent the kernel and bias transformed from {W,μ,σ,β} .


(3) $${W^{\prime }}_{i,:,:,:}=\frac{\gamma_{i}}{\sigma_{i}}∼{\text{W}}_{i,:,,:,:},{b^{\prime }}_{i}=-\frac{\mu_{i}\gamma_{i}}{\sigma_{i}}+\beta_{i}$$

Easily verified:


(4) $$bn{\left(M*-\text{W,}\mu ,\sigma ,\gamma ,\beta \right)}_{:,i,;,:}={\left(M*-W\prime \right)}_{:,,i,:;}+b_{i}^{\prime }$$

In the second step, the convolutional operators across all three branches are consolidated into the form of 3×3 convolution kernels and biases, which are aggregated to yield the final outcome on the main branch. We need to add the three bias vectors to get the final bias and get the final 3×3 convolution by adding the 1×1 convolution to the center of the 3×3 convolution kernel.

### 3.2 Partial convolution

**Figure 4:**
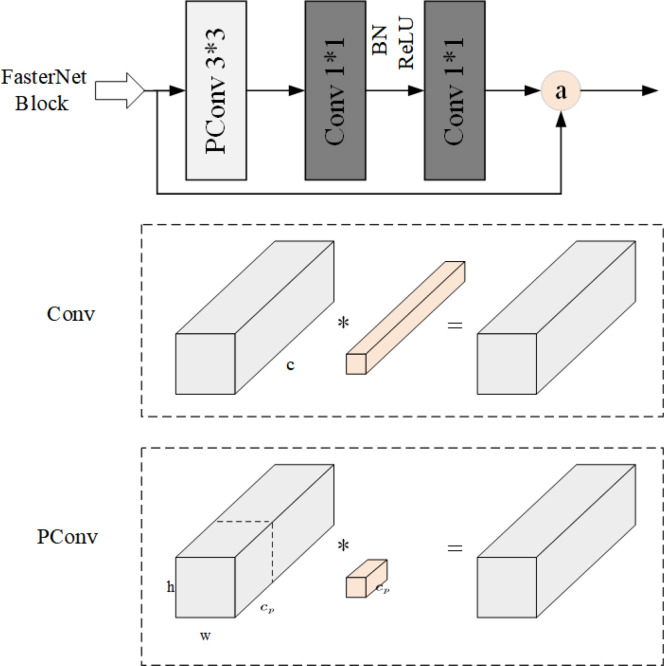
FasterNet Block structure.

The PConv-based FasterNet represents a novel neural network designed to achieve enhanced processing speed on devices while maintaining accuracy in target detection. It comprises four layered stages, interspersed with FasterNet Blocks. Each FasterNet Block consists of a PConv layer followed by two Convolutional layers, collectively constituting the FasterNet Block, as illustrated in **Figure 4**.

PConv leverages the redundancy inherent in the feature map by using selectively making use of ordinary convolution completely to a subset of the enter channels, whilst leaving the rest untouched. This strategy correctly mitigates computational redundancy. PConv calculates the first or closing consecutive $c_{p}$ channels as representatives of the complete feature map, with Floating Point Operations (FLOPs) computed as follows:


(5) $$h*w*k^{2}*c_{p}^{2}$$

The FLOPs for regular convolution are computed as follows:


(6) $$h*w*k^{2}*c^{2}$$

When the ratio is $r=c_{p}/c=1/4$ , the FLOPs of PConv quantity to solely 1/16th of these incurred with the aid of ordinary Convolution, main to a tremendous discount in the model’s complexity. In order to absolutely leverage the facts from all channels, ordinary convolution is appended to PConv.

### 3.3 Efficient multi-scale attention

**Figure 5:**
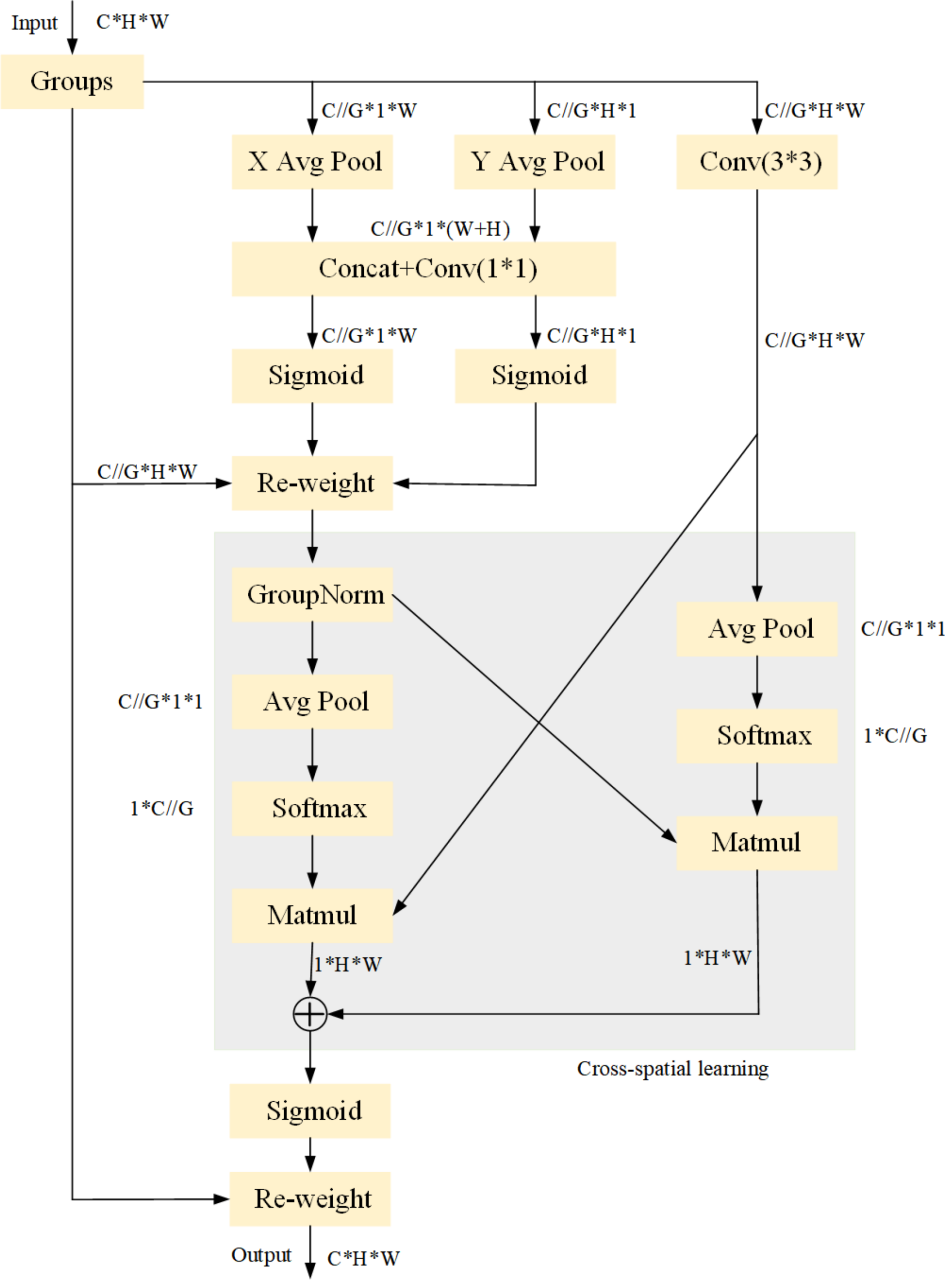
EMA structure.

The EMA mechanism represents an enhancement over Channel Attention (CA). CA utilizes one-dimensional global average pooling along the x and y dimensional directions to capture long-range interactions in space across different dimensions. However, it overlooks the significance of interactions between locations across space. This study ensures that the spatial semantic features are distributed uniformly by transforming some channels into batch dimensions and grouping the channel dimensions into several sub-features. **Figure 5** illustrates the EMA’s configuration.

For any given input feature mapping $X\in R^{C*H*W}$ , in order to support the learning of various semantics, the EMA splits X into G sub-features along the channel dimension directions. The following formula can be used to determine the grouping style.


$$X=[X_{0},X_{i},\ldots ,X_{G-1}],X_{i}\in R^{C//G*H*W}$$

Three parallel paths are used by EMA in the first phase to extract grouped feature maps; two of these routes are in the 1×1 branch, while the third is in the 3×3 branch. Stacking a single 3×3 convolution in the 3×3 branch for capturing multi-scale features, while two 1D global average pooling procedures are used in the 1×1 branch to encode channels across several spatial directions. In parallel, the input tensor is redefined as C//G*H*W and the group G results are reintegrated into the batch dimension. A Sigmoid function is used to account for the 2D distribution of the linear convolution after the output of the 1×1 convolution is broken down into two vectors.

Two tensors are added in the cross-space learning phase: one from the 1×1 branch’s output and another from the 3×3 branch’s output. 2D pooling is used to encode information into the 1×1 branch’s output. Furthermore, before the channel features’ joint activation process, the output of the last branch is reshaped to the matching shape. The following is the expression used to formulate the 2D pooling function:


(7) $$z_{c}=\frac{1}{H\times W}\sum_{j}^{H}\sum_{i}^{W}x_{c}(i,j)$$

Softmax is utilized to adopt a linear transformation to the 2D global average pooling’s output in order to guarantee computational efficiency. The output is then subjected to matrix dot product operation, which produces the first spatial attention map. On the third branch, 2D global average pooling is utilized to encode the global spatial information in that branch and matrix multiplication is performed with the third branch before the 1×1 branch activation mechanism. Consequently, the second spatial interest map is generated, retaining particular spatial region information.

The output feature maps inside every crew are then computed as an aggregation of the two generated spatial interest weight values, culminating in a Sigmoid characteristic software taking pictures the world context of every pixel.

## 4 Results and analyses

### 4.1 Experimental environment

To check the efficacy of the more advantageous model, we set up an experimental platform using Windows 10 as the working machine and PyTorch as the deep studying framework. The RT-DETR served as the foundational community model. The unique configuration of the experimental surroundings is outlined in **Table 1**.

**Table 1:** Configuration of the experimental platform.

Classification	Configuration
System Environment	Windows 10
GPU	GeForce RTX 3050 Ti
Framework	Pytorch1.13.1
Programming Language	Python 3.8

Consistent configuration parameters were maintained throughout the experiment’s training phase, with details provided in **Table 2**.

**Table 2:** Configuration parameters for process.

Parameters	Value
Learning Rate	0.01
Image Size	640*640
Batch Size	4
Epoch	100
Momentum	0.9
Weight Decay	0.0001
Optimizer	AdamW

### 4.2 Datasets and assessment indicators

The dataset utilized in this investigation comprised plant images sourced from online repositories. To annotate the diverse plant images, rectangular bounding boxes were manually labeled using the Labeling library, with annotations stored in XML format and subsequently converted into TXT format. Specifically, the dataset comprises 9720 images encompassing five categories: ripe banana, ripe tomato, overripe banana, unripe banana, and unripe tomato. These images were partitioned into training, validation, and test sets in an 8:1:1 ratio.

To facilitate an objective evaluation of the plant detection model’s performance, several evaluation metrics were employed, including FPS, GFLOPs, and mAP. FPS denotes the number of images detected per second by the target detection network; a higher FPS indicates better real-time performance. GFLOPs are used to measure the complexity of the model, which is proportional to the number of parameters. The mAP, utilized to gauge the accuracy of the model, was calculated using Equation 8.


(8) $$mAP=\frac{\sum_{i=1}^{N}AP_{i}}{N}$$

N denotes the number of categories, and $AP_{i}$ denotes the average precision of the ith category. average precision can be obtained by plotting the Precision-Recall (PR) curve and calculating the integral area of the curve. mAP@0.5 is calculated as the mean value of the model’s AP in each category at an IoU threshold of 0.5.

### 4.3 Ablation experiment

To ascertain the accuracy of the model, five sets of ablation experiments were conducted in THIS study, with the results presented in **Table 3**. The addition of the EMA mechanism following the Stem block, facilitating the extraction of image feature information across spatial and locational dimensions, led to a 1.81% enhancement in model accuracy. However, this augmentation was accompanied by an increase in model complexity.

**Table 3:** Results of ablation experiments.

Rep Block	PConv	EMA	mAP@0.5(%)	FPS	GFLOPs	Model Size(M)
			93.10	33.4	57.0	38.6
		√	94.91	27.6	59.1	38.9
√			95.62	33.3	57.1	38.8
√	√		95.11	32.4	49.5	32.9
√	√	√	96.00	26.7	51.5	33.1

Reconstructing the backbone portion of RT-DETR using Rep Block resulted in an improved average accuracy, with a mean Average Precision mAP of 95.62%, without significant alterations in model size, detection speed, or computational complexity. Substituting partial convolution for Rep Block’s conventional convolution significantly reduced the computational complexity and model size. Nevertheless, this modification corresponded to a decrease in model accuracy by 0.51 percentage points. When the enhanced Rep Block module and EMA were jointly incorporated into the backbone network, the model accuracy increased from 93.10% to 96.00% compared to the RT-DETR model. Moreover, there was a substantial reduction in model size and parameter counts, facilitating the efficient deployment of subsequent models.

**Figure 6:**
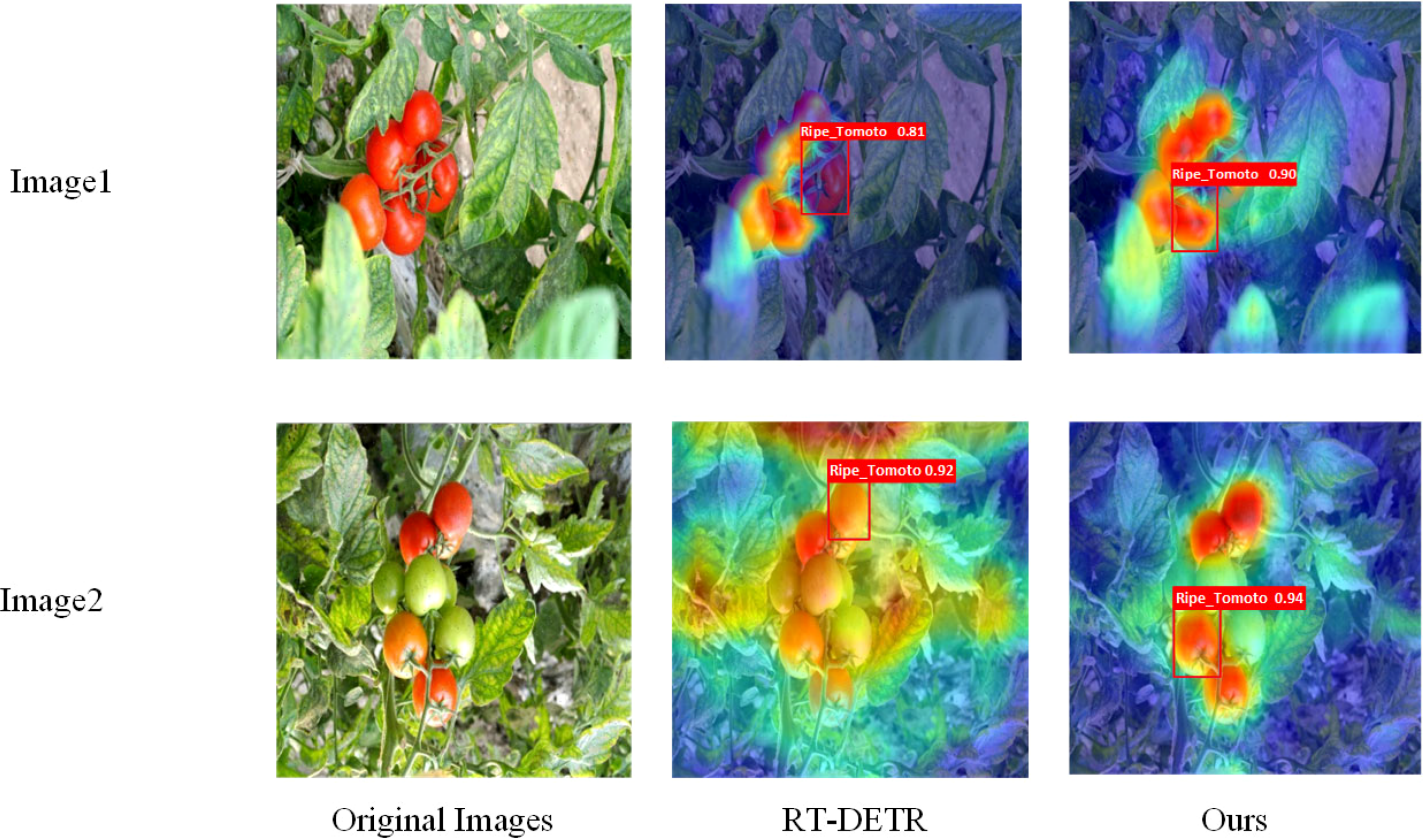
Heat map visualization of the improved model versus the original model.

Overall, the improved version of RT-DETR, integrating Partial Convolution (PConv), Rep Block, and the EMA Attention Module, surpassed the original model in the fact of detection accuracy, computational complexity, and model saved size. Employing the same dataset and training parameters, our model achieved a 2.9% enhancement in mAP@0.5, a 14.2% reduction in model size, and a 9.6% reduction in computational complexity. **Figure 6** illustrates the visual comparative effect of the heat maps generated by the improved RT-DETR model and the original model.

**Figure 6** illustrates a visual comparison of the thermographic results obtained from the improved RT-DETR model and the original model. The left column presents the original images, while the middle and right columns display the detection results produced by the RT-DETR model and the proposed method, respectively. The heat maps superimposed on the images represent the areas of focus during the detection process. Specifically, warmer colors (red and yellow) correspond to regions with higher attention or confidence levels, whereas cooler colors (blue and green) denote areas with lower attention. The bounding boxes outline the detected ripe tomatoes, with the text inside each box indicating the corresponding confidence levels for the detections.

### 4.4 Model performance experiments

**Figure 7:**
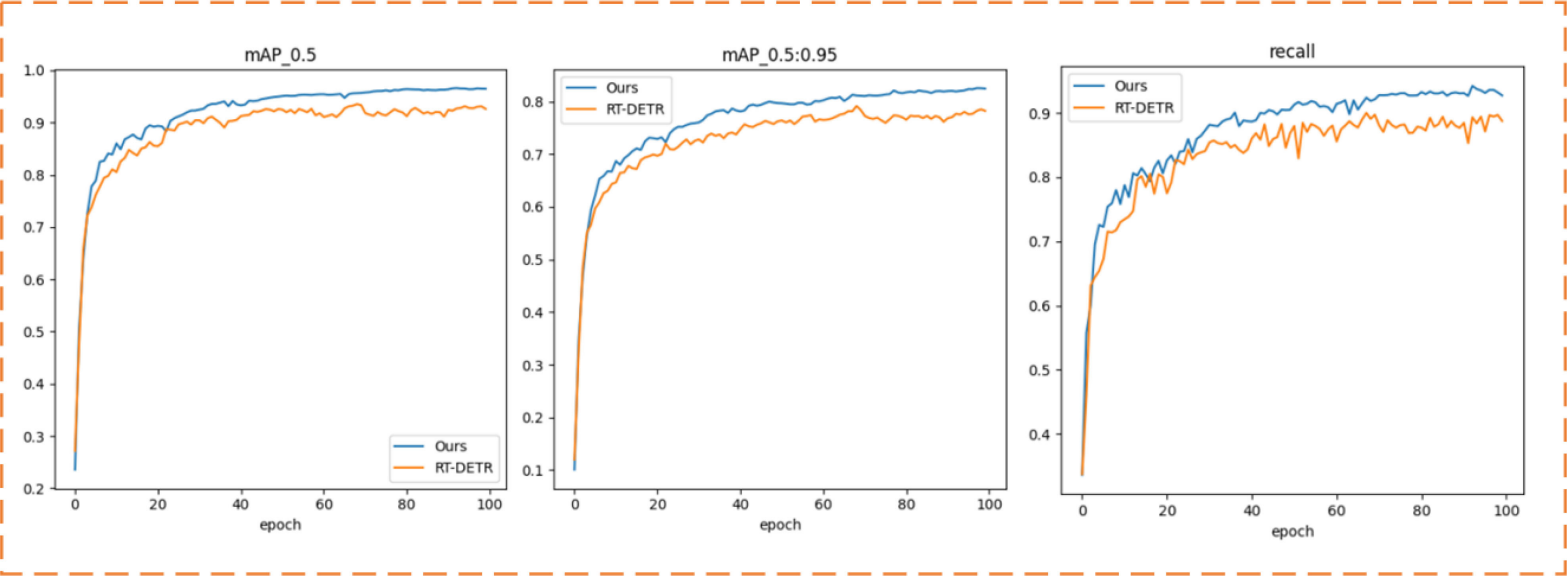
mAP curve, recall curve.

**Figure 8:**
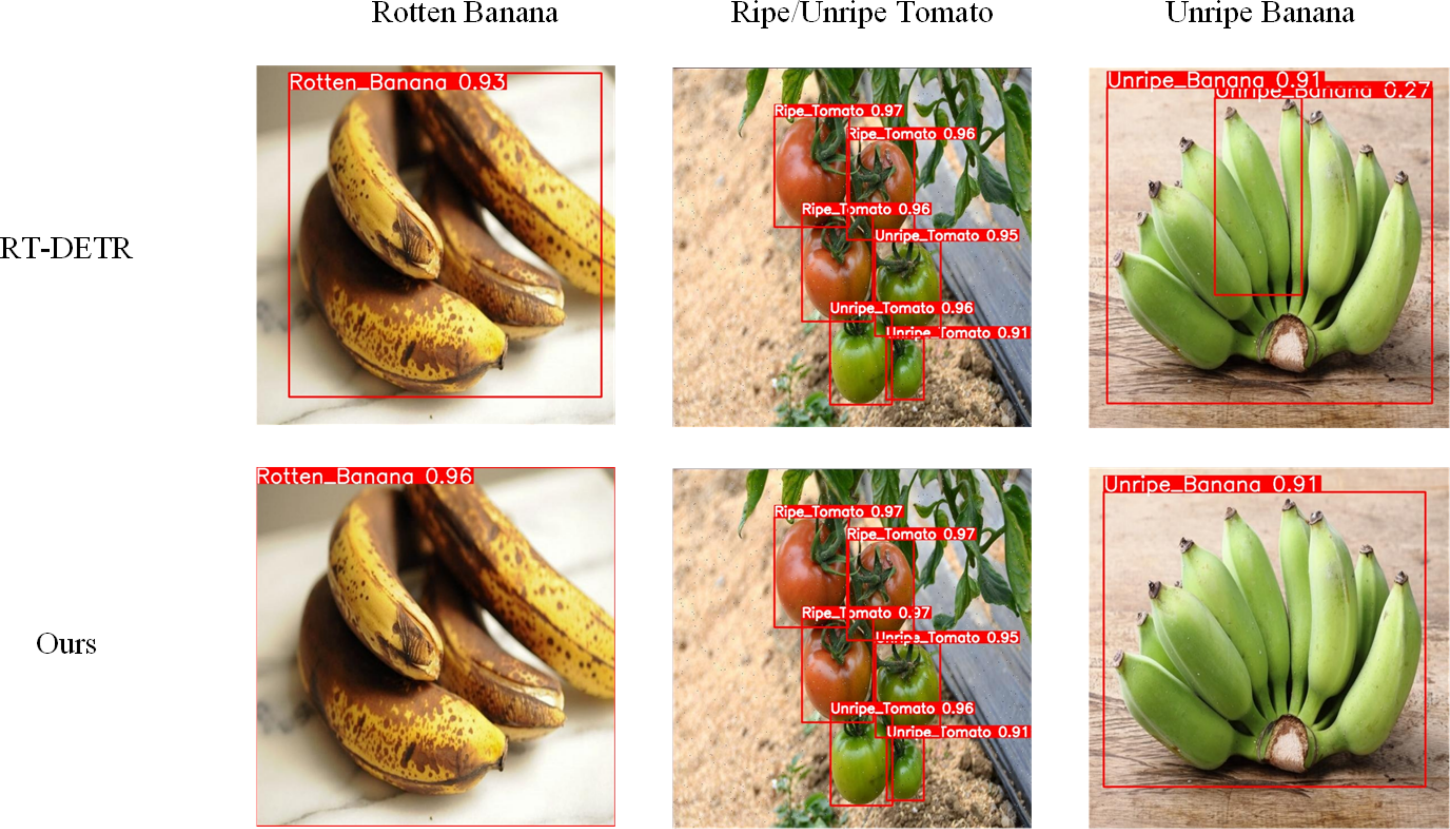
Comparison of test results before and after modification.

The interpretability of deep learning models’ performance holds paramount importance, particularly in domains such as target detection and instance segmentation. In this segment of the experiment, we selected the improved RT-DETR model alongside the RT-DETR model as the validation models, analyzing the curve comparisons of the two models, as depicted in **Figure 7**, and assessing the picture ground detection performance, as shown in **Figure 8**.

The mAP@0.5, mAP@0.5:0.95, and recall curves presented in **Figure 7** offer intuitive evidence of the superior performance of the improved RT-DETR model: Our model’s accuracy starts to level off around 50 rounds of training. It is also significantly more accurate than the original RT-DETR model during the training process.

The highlighted area within the square region of **Figure 8** delineates the types of plants recognized by the model, with accompanying numerical values denoting the confidence levels of detection. As per the experimental outcomes, in comparison to the original model, the improved model exhibits enhancements in the confidence level during ripe banana detection. Moreover, in the detection of occluded tomatoes and unripe bananas, the improved model demonstrates a notably higher detection proficiency for these objects. This observation is attributed to the integration of the RepBlock, which enhances the network’s sensitivity and adaptability to target detection, and the inclusion of EMA and PConv in the model’s optimization, enhancing its feature recognition capabilities. When testing Unripe Banana, the improved model appeared to have fewer anchor frames. Overall, the improved model significantly enhanced the model’s detection ability.

**Figure 9:**
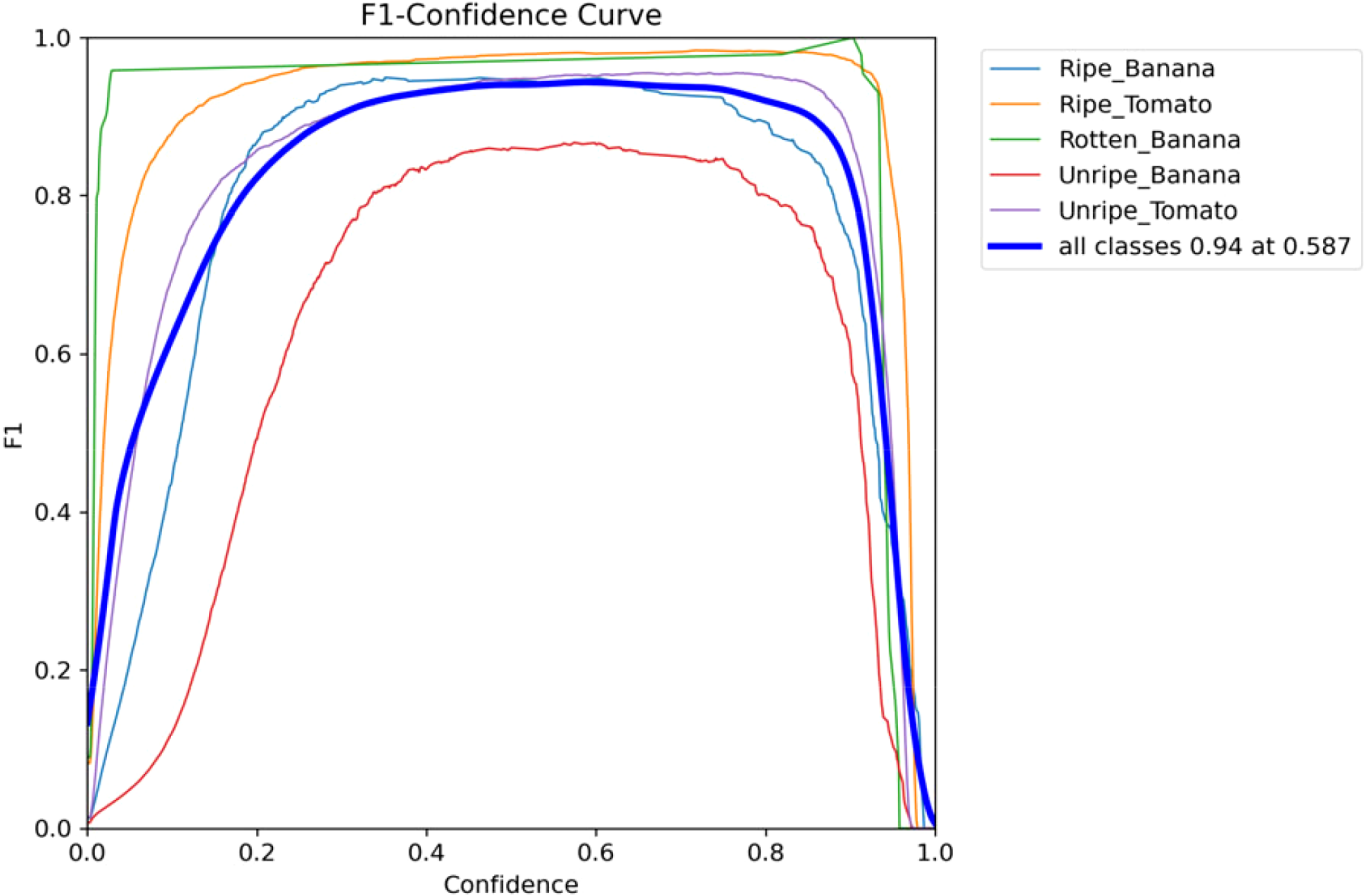
F1 confidence curve for the improved model.

The F1 score (Chicco and Jurman, 2020) serves as a widely adopted metric for assessing object detection models, representing the weighted average of precision and recall. **Figure 9** illustrates the F1-confidence curves for the improved RT-DETR.

1. A high peak F1 score and a maximum F1 score close to 1 signify the optimal performance of the model.
2. The curves exhibit broad curves and flat peaks, indicating that the F1 scores remain stable across different thresholds. Thus, the improved model demonstrates robustness.
3. The area under the curve, which summarizes the performance for all thresholds, is larger. A larger area signifies a superior model. In this scenario, the model excels in the detection of rotten bananas and ripe tomatoes.

### 4.5 Comparison of different models

In order to evaluate the performance of the enhanced model, this study conducted comparison experiments between the improved model and various widely used RT-DETR series models (RT-DETR-R18, RT-DETR-R34, RT-DETR-R50, RT-DETR-X, RT-DETR-L), YOLOv8n, which were conducted on the same dataset and the same experimental conditions.

As shown in the experimental results in **Table 4**, the improved model obtained 96.00% mAP, which is significantly higher than the first five tested models. The accuracy of this model is not much different from that of RT-DETR-X, but the number of parameters of the improved model is only 1/6 of the original model, which shows commendable computational volume and computational complexity. Compared with the YOLOv8n model, its mAP increased by 7.67 percentage points. These results demonstrate that the improved model is able to maintain the computational volume while still exhibiting high accuracy.

**Table 4:** Results of model comparison experiments.

Model Name	Input shape	mAP_0.5(%)	GFLOPs	Parameters
RT-DETR-R18	640*640	93.10	57.0	19974480
RT-DETR-R34	640*640	93.88	89.1	31227972
RT-DETR-R50	640*640	94.26	129.9	42118508
RT-DETR-L	640*640	94.35	103.8	32148396
RT-DETR-X	640*640	95.74	222.8	65632092
YOLOv8	640*64	88.31	8.1	3006623
Ours	640*640	96.00	51.5	16996720

## 5 Discussion

**Figure 10:**
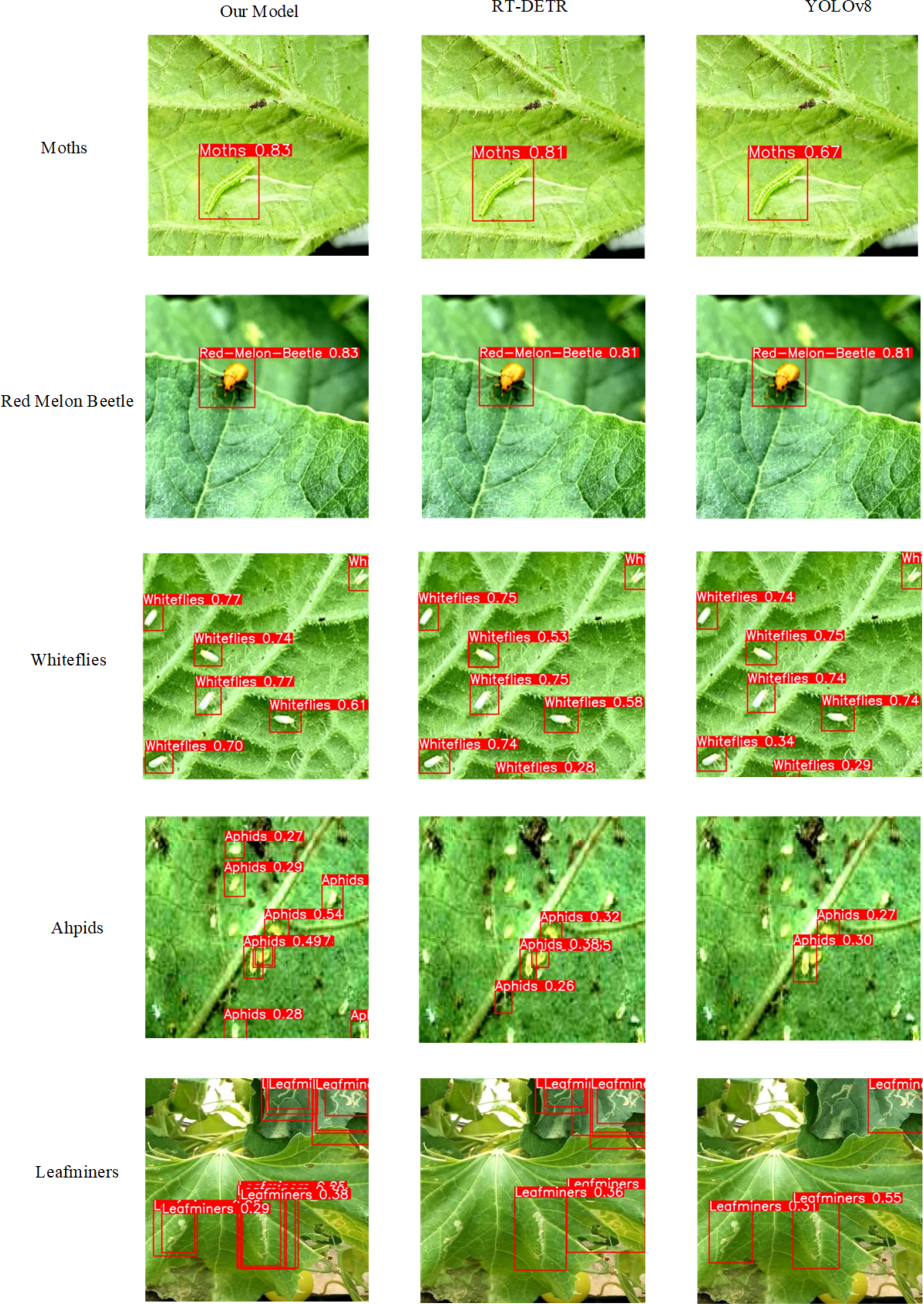
Detection results of different models in pest detection.

The model proposed in this paper can be applied not only to plant maturity detection but also to other fields. To verify the generality of the model, we obtained a dataset of plant pests from the public website roboflow, which contains a total of five categories of pests: Aphids, Leafminers, Moths, Red-Melon-Beetle, and Whiteflies. we trained the improved model, RT-DETR model, and YOLOv8 model on this dataset, and its detection results are shown in **Figure 10**.

The results show that: our model is better than the other models in the detection of Moths, Red-Melon-Beetle pests, and its detection accuracy has been improved a little; when detecting Whitflies pests, there is not much difference in the effectiveness of the models; and in the detection of Aphids and Leafminers, the standard RT-DETR model and the YOLOv8 model not only have low detection accuracy, but also have obvious leakage detection, the improved RT-DETR model obviously reduces the leakage detection rate and also improves the detection accuracy. Through this experiment we verified the generality of the model.

## 6 Conclusion

Plant maturity detection holds pivotal importance in advancing automated harvesting technologies. In this study, an improved version of a plant detection model based on RT-DETR-R18 was developed. The methodology initially integrates EMA mechanism into the backbone network to enhance information extraction pertaining to space and location. Subsequently, the Rep Block reconstructs the backbone structure of the original model, thereby augmenting model accuracy. Furthermore, the PConv module replaces ordinary convolution, leading to reduced complexity and model size.

Experimental consequences point out the model’s suitability for various detection scenarios. The expanded RT-DETR mannequin substantially outperforms different mainstream RT-DETR household detection fashions (RT-DETR-R18, RT-DETR-R34, RT-DETR-R50, RT-DETR-L, and RT-DETR-X) by means of accomplishing detection accuracies of 2.9%, 2.12%, 1.74%, 1.65%, respectively, and 0.26%. Moreover, the more desirable mannequin enhances detection accuracy whilst lowering mannequin parameters and computational complexity. Compared to the original model, the model size is reduced by 5.5% and the computational complexity is reduced by 9.6%. This makes the improved model suitable for scenarios with memory and computational constraints, such as embedded devices.

In future lookup endeavors, we intend to diversify information series by way of incorporating extra scenes of tomato and banana images, consisting of tomato pics obscured via leaves and banana snap shots in complicated environments. Additionally, we intention to accumulate extra data, encompassing parameters like wavelength and vibration, to similarly combine photograph facts and decorate the model’s reliability.

## Data Availability

The original contributions presented in the study are included in the article/supplementary material. Further inquiries can be directed to the corresponding author.
